# Persistence of neutralizing antibodies a year after SARS‐CoV‐2 infection in humans

**DOI:** 10.1002/eji.202149535

**Published:** 2021-10-08

**Authors:** Anu Haveri, Nina Ekström, Anna Solastie, Camilla Virta, Pamela Österlund, Elina Isosaari, Hanna Nohynek, Arto A Palmu, Merit Melin

**Affiliations:** ^1^ Department of Health Security Finnish Institute for Health and Welfare Helsinki Finland; ^2^ Department of Public Health and Welfare Finnish Institute for Health and Welfare Helsinki Finland

**Keywords:** IgG antibodies, neutralizing antibodies, SARS‐CoV‐2, seroprevalence, variants of concern

## Abstract

Most subjects develop antibodies to SARS‐CoV‐2 following infection. In order to estimate the duration of immunity induced by SARS‐CoV‐2 it is important to understand for how long antibodies persist after infection in humans. Here, we assessed the persistence of serum antibodies following WT SARS‐CoV‐2 infection at 8 and 13 months after diagnosis in 367 individuals. The SARS‐CoV‐2 spike IgG (S‐IgG) and nucleoprotein IgG (N‐IgG) concentrations and the proportion of subjects with neutralizing antibodies (NAb) were assessed. Moreover, the NAb titers among a smaller subset of participants (*n* = 78) against a WT virus (B) and variants of concern (VOCs): Alpha (B.1.1.7), Beta (B.1.351), and Delta (B.1.617.2) were determined. We found that NAb against the WT virus persisted in 89% and S‐IgG in 97% of subjects for at least 13 months after infection. Only 36% had N‐IgG by 13 months. The mean S‐IgG concentrations declined from 8 to 13 months by less than one third; N‐IgG concentrations declined by two‐thirds. Subjects with severe infection had markedly higher IgG and NAb levels and are expected to remain seropositive for longer. Significantly lower NAb titers against the variants compared to the WT virus, especially after a mild disease, suggests reduced protection against VOCs.

## Graphical abstract

A year after WT SARS‐CoV‐2 infection high seropositivity rate was observed: 89% of subjects had persisting neutralizing antibodies and up to 97% had anti‐spike IgG antibodies. Compared to the WT virus, neutralizing antibody titers were reduced for variants of concern Alpha, Beta, and Delta.

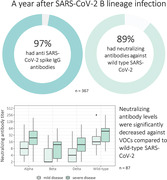

## Introduction

Infection with Severe acute respiratory coronavirus 2 (SARS‐CoV‐2) induces antibodies in most subjects to viral nucleoprotein (N) and spike (S) glycoprotein (1). Neutralizing antibodies (NAb) against SARS‐CoV‐2 target the receptor‐binding domain (RBD) of the S protein and sterically interfere with the binding of the viral S protein and the host's angiotensin‐converting enzyme 2 (2, 3). NAb levels are highly predictive of protection against infection and clinical disease (4) and detectable NAb have been reported to persist in most subjects at least 6 to 12 months after infection (5, 6, 7, 8, 9, 10, 11, 12, 13). Previous findings suggest that neutralizing activity against the SARS‐CoV‐2 is mediated particularly by IgG1 and IgA antibodies (14, 15). However, as the concentration of anti‐SARS‐CoV‐2 IgA antibodies has been shown to decline rapidly following infection (16, 17, 18), long‐term neutralization is thus driven by IgG antibodies to the spike protein (16).

SARS‐CoV‐2 is constantly mutating yet most changes have little or no impact on its virulence (19). However, some changes are causing concerns regarding disease severity, viral transmissibility, and potential escape from natural and vaccine‐induced immunity (20). The World Health Organization (WHO) in collaboration with an international network of experts has characterized the variants of concern (VOC) (https://www.who.int/en/activities/tracking‐SARS‐CoV‐2‐variants/). Reduced NAb levels as compared to the WT virus have been shown against VOCs, especially against the Beta variant, both after vaccination (13, 21–23) and 9 (13) and 12 months (12) after infection. A similar reduction in NAb titers has also been reported against the Delta variant from convalescent sera collected 3–12 months post symptoms or after vaccination (24, 25).

Previous infection with SARS‐CoV‐2 has shown to induce effective immunity and protection against reinfections in most individuals (26, 27). In animal studies, a protective antibody titer against SARS‐CoV‐2 infection has been suggested to be low (28, 29). Higher IgG antibody levels against SARS‐CoV‐2 among health care workers within three months after vaccination were found to be associated with lower infectivity (30). However, a protective threshold for humans is still under debate and subject to the standardization of serological methods. The accumulating research data on the persistence of antibodies after natural infection, and NAbs in particular, will provide important insight into estimating for how long antibodies induced by Coronavirus disease 2019 (COVID‐19) vaccination can be expected to persist and provide protection against emerging SARS‐CoV‐2 variants. In this study, we investigated the antibody persistence up to 14 months after natural SARS‐CoV‐2 infection and assessed the potential cross‐protection by comparing the NAb levels of WT virus (B lineage) to three VOC strains Alpha (B.1.1.7), Beta (B.1.351), and Delta (B.1.617.2).

## Results

### Persistence and kinetics of SARS‐CoV‐2 antibodies

**Figure 1:**
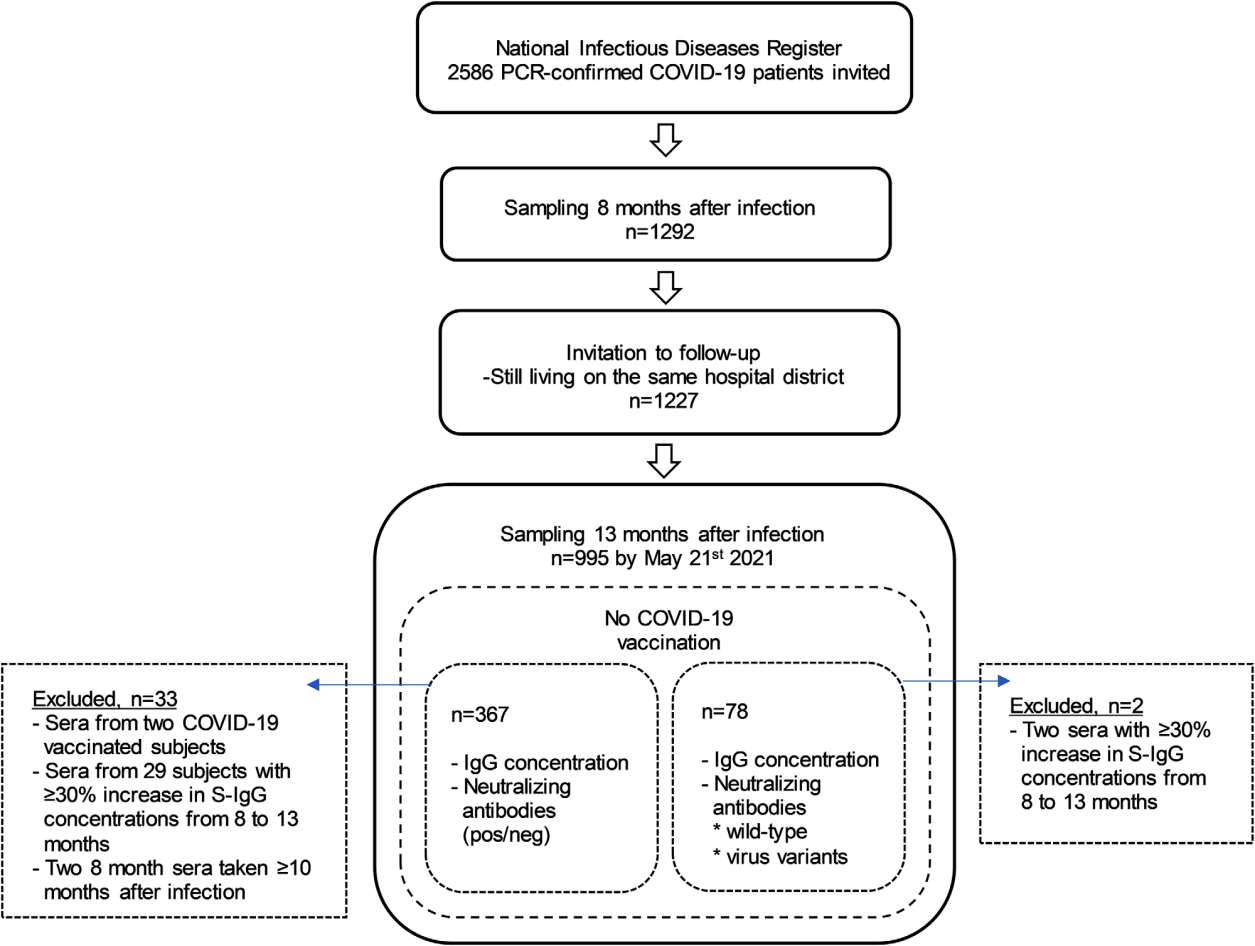
The study flow chart showing the selection of serum samples of the study participants for the determination of antibody concentration and neutralizing antibodies 8 and 13 months after infection.

**Figure 2:**
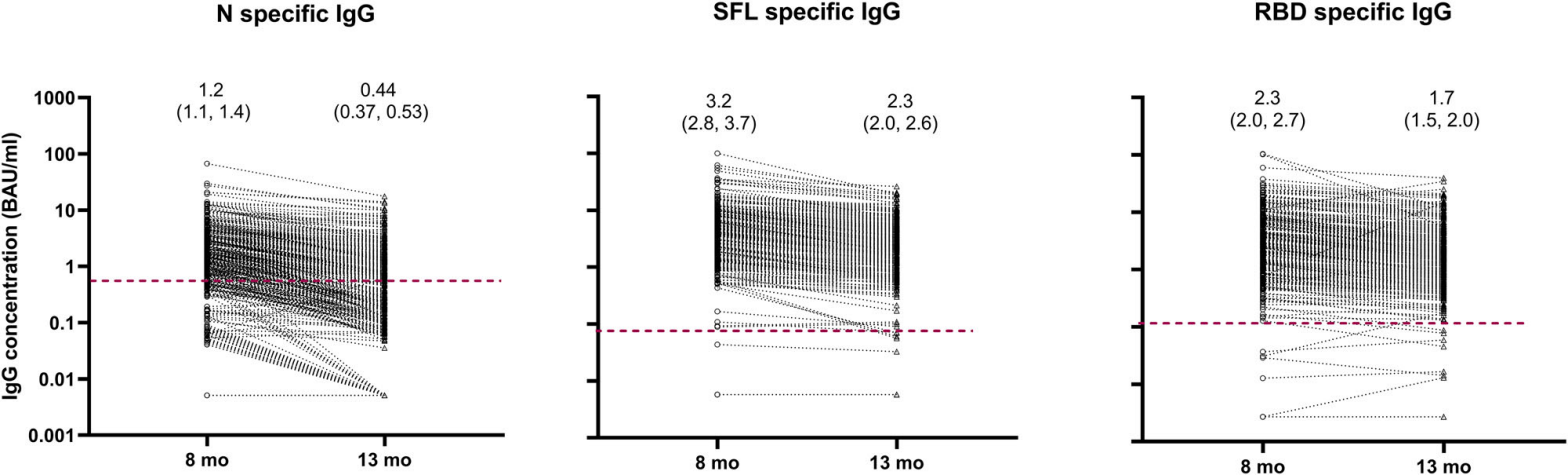
Nucleoprotein (N), and spike protein (SFL, RBD) specific IgG concentrations (BAU/ml) with geometric mean concentrations (95% CI) at 8 and 13 months after infection, *n* = 367 subjects. FMIA specific cut‐off for seropositivity is indicated by a dashed red line. Each sample was tested as technical duplicates in each experiment and the experimental precision was confirmed by two control samples in each independent experiment.

We first assessed the persistence of NAb and serum IgG antibodies specific to SARS‐CoV‐2 Spike full length (SFL)‐IgG, RBD‐IgG, and N‐IgG at 8 months following SARS‐CoV‐2 infection. We found that 89% (1148/1292) of the subjects had NAb against the WT virus, 96% (1240/1292) had antibodies to SFL and RBD (S‐IgG) and 66% (846/1292) had N‐IgG. We further assessed the persistence of NAb and IgG antibodies a year after SARS‐CoV‐2 infection by randomly selecting 367 of 652 subjects who had not received a SARS‐CoV‐2 vaccination of the 995 subjects who participated at both time points (Fig. 1). Participant demographics and clinical characteristics for the selected cohort were similar to the overall cohort (Table 1). NAb, S‐IgG, and N‐IgG antibodies were detected in 91%, 98%, and 67% of subjects in the selected cohort at 8 months after infection, respectively (Table 2). One year after infection the proportion of positive samples was still high for NAb and S‐IgG (89% (326/367) and 97% (356/367)), respectively, but had decreased to 36% (132/367) for N‐IgG. The mean IgG concentrations decreased significantly (*p* < 0.001) for SFL‐IgG, RBD‐IgG, and N‐IgG from 8 months (3.2, 2.3, 1.2 binding antibody unit concentrations (BAU)/ml) to 13 months (2.3, 1.7, 0.44 BAU/ml, respectively) after infection. The decrease in mean IgG concentration was more notable (‐63%) for N‐IgG compared to SFL‐IgG (‐28%) or RBD‐IgG (‐26%) (Fig. 2).

**Table 1:** Demographics and clinical characteristics of study participants in the study cohorts at 8 and 13 months after infection

	8 months participants	13 months participants	Study Cohort	Sub Cohort
**N**				
**8 months**	1292	N/A	367	N/A
**13 months**	N/A	995	367	78
**Gender**				
**Male n (%)**	520 (40%)	386 (39%)	159 (43%)	40 (51%)
**Female n (%)**	772 (60%)	609 (61%)	208 (57%)	38 (49%)
**Age at diagnosis** **(median, range)**				
**<60y**	45.1 (17.3‐59.9)	47.5(17.6‐59.9)	45.9 (17.7‐59.9)	51.6 (19.0‐59.7)
**>60y**	65.1 (60.0‐94.3)	65.4 (60.0‐95.6)	63.3 (60.0‐79.0)	63.0 (60.0‐81.3)
**All**	50.0 (17.3‐94.3)	52.5 (17.6‐95.6)	48.8 (17.7‐79.0)	59.4 (19.0‐81.3)
**Time (mo) after diagnosis at sampling**				
**8 months**	7.6 (5.9‐9.9)	N/A	7.6 (6.1‐9.7)	N/A
**13 months**	N/A	12.7 (11.7‐14.3)	12.7 (11.9‐14.0)	13.0 (12.2‐13.6)
**Disease severity**				
**Severe**	190 (15%)	149 (15%)	47 (13%)	39 (50%)
**Mild**	1102 (85%)	846 (85%)	320 (87%)	39 (50%)

**Table 2:** Number and proportion of positive samples for spike protein IgG (S‐IgG) and neutralizing antibodies (NAb) by disease severity, age and gender of the participants 8 and 13 months after infection, n=367

			S‐IgG positive n/n (%)				NAb positive (wt) n/n (%)			
Disease severity	Age (years)	Gender	8 months		13 months		8 months		13 months	
**Severe**	≥60	M	16/16	(100)	16/16	(100)	16/16	(100)	16/16	(100)
		F	18/18	(100)	18/18	(100)	17/18	(94)	18/18	(100)
	<60	M	6/6	(100)	6/6	(100)	6/6	(100)	6/6	(100)
		F	7/7	(100)	7/7	(100)	7/7	(100)	7/7	(100)
**Mild**	≥60	M	120/122	(98)	117/122	(96)	105/122	(86)	99/118	(84)
		F	166/171	(97)	165/171	(97)	159/171	(93)	151/171	(88)
	<60	M	15/15	(100)	15/15	(100)	15/15	(100)	14/15	(93)
		F	12/12	(100)	12/12	(100)	10/12	(83)	12/12	(100)

### Effect of disease severity, age, and gender on SARS‐CoV‐2 antibodies

**Figure 3:**
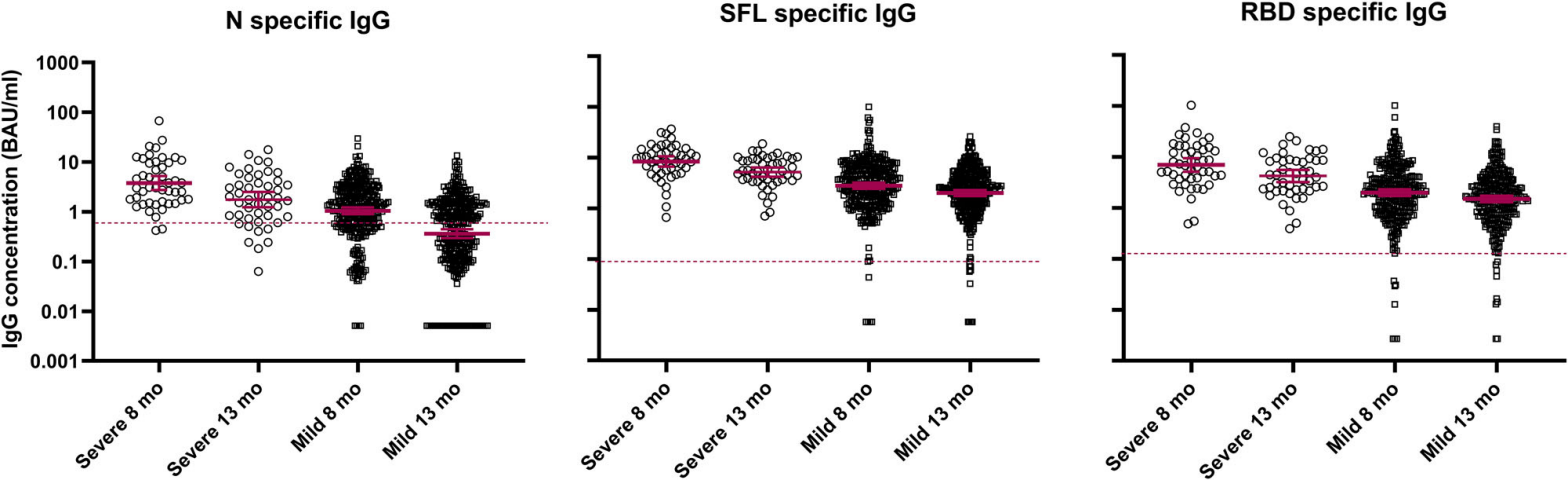
Distribution and the geometric mean of IgG concentrations (BAU/ml and 95% CIs) for nucleoprotein (N specific IgG), and spike protein (SFL and RBD specific IgG) in subjects 8 and 13 months after severe (*n* = 47 subjects) or mild (*n* = 320 subjects) infection. FMIA specific cut‐off for seropositivity is indicated by a dashed red line. Each sample was tested as technical duplicates in each experiment and the experimental precision was confirmed by two control samples in each independent experiment.

We observed higher mean N‐IgG, SFL‐IgG, and IgG‐RBD concentrations in subjects who had recovered from severe disease than in those with mild disease 8 months after infection (*p* < 0.001; Fig. 3). The difference was 2.0‐ to 7.4‐fold, depending on the age group, and persisted for at least 13 months after infection (Fig. 3, Table 3). The proportion of seropositive subjects remained high for S‐IgG and NAb (100%) and relatively high for N‐IgG (67%) a year after severe infection, compared to 97%, 87%, and 32%, respectively, of those with a milder infection. A higher proportion (33%) of subjects in the elderly age group (≥60 years of age) had been hospitalized compared to the younger age groups (13% of 40 to 59 years and 6% of those 17 to 39 years of age). Elderly subjects (≥60 years of age) with mild infection had similar levels of S‐IgG antibodies (Table 3) and an equally high proportion of them had NAb compared to younger subjects with mild infection. N‐IgG concentrations were, however, higher among ≥60‐year old subjects than in subjects <60 years of age with a mild disease at 8 and 13 months after infection (*p* < 0.01). We could not demonstrate any difference in N‐, SFL‐, or RBD‐IgG concentrations between males and females at 8 or 13 months after infection.

**Table 3:** Geometric mean IgG concentrations, GMC [95% CI], expressed as BAU/ml for nucleoprotein (N), spike proteins (SFL and RBD) at 8 and 13 months after COVID‐19 infection per age group and disease severity. Significantly higher (Kruskal‐Wallis test, p<0.05) IgG concentrations in subjects with severe as compared to mild disease within age groups are shown in bold

Disease	Age (years)n	N‐IgGGMC [95% CI]		RBD‐IgGGMC [95% CI]		SFL‐IgGGMC [95% CI]	
		8 months	13 months	8 months	13 months	8 months	13 months
**Mild**	**17‐39** n=101	0.71 [0.40–1.0]	0.23 [0.0061–0.46]	1.7 [0.87–2.5]	1.5 [0.85–2.1]	2.5 [1.1–4.0]	2.0 [1.4–2.6]
	**40‐59** n=192	1.2 [0.74–1.6]	0.41 [0.18–0.64]	2.0 [0.68–3.4]	1.5 [0.83–2.1]	2.8 [1.4–4.1]	1.9 [1.4–2.5]
	**≥60** n=27	2.1 [0.32–3.9]	0.81 [‐0.29 –1.9]	3.0 [1.1–4.8]	1.9 [‐0.70–4.5]	4.0 [1.9–6.1]	2.6 [1.1–4.2]
**Severe**	**17‐39** n=6	2.9 [‐1.9–7.7]	**1.7** **[‐0.33–3.7]**	8.4 [‐33–50]	4.6 [0.19–9.0]	6.9 [0.35–14]	5.1 [0.82–9.4]
	**40‐59** n=28	**3.9** **[‐0.96–8.7]**	**1.8** **[0.29–3.2]**	**6.2** **[3.5–8.8]**	**4.0** **[1.8–6.2]**	**7.6** **[5.2–10]**	**4.7** **[3.2–6.3]**
	**≥60** n=13	4.1 [‐1.4 –9.5]	1.8 [‐0.91–4.5]	8.4 [1.4–15]	4.5 [0.89–8.1]	**11.4** **[5.2–18]**	6.4 [4.1–8.7]

### Comparison of NAb titers between a WT virus and three VOCs

A smaller age‐ and gender‐matched subset of participants (*n* = 78) of 13‐month samples was randomly selected for NAb titration due to the laborious live‐virus microneutralization test (MNT). The samples were re‐analyzed against a WT virus isolated in Finland during 2020 and three VOCs (Alpha, Beta, and Delta) isolated in Finland during 2021. The samples to be included in the NAb titration were selected based on a seropositive result (NAb titer ≥6) in the screening test.

Within the whole cohort (n = 78), NAb titers were significantly lower for all VOCs (*p* < 0.0001, Kruskal‐Wallis test) compared to WT virus. This decrease in geometric mean titers (GMT) was more notable for the Beta (‐77%) and Delta (‐69%) variants than for the Alpha variant (‐42%) (Table 4). NAb titers for all VOCs correlated well with WT virus titers, yet a more pronounced correlation was seen for the Alpha and Delta variants and lower for the Beta variant (Supporting information Fig. 1).

**Table 4:** Geometric mean IgG concentrations, GMC [95% CI] expressed as BAU/ml for nucleoprotein (N) and spike proteins (SFL and RBD) and geometric mean titers, GMT [95% CI] of neutralizing antibodies (NAb) against wild‐type (wt) virus and three variants of concern Alpha (B.1.1.7), Beta (B.1.351) and Delta (B.1.617.2) 13 months after infection (n=78)

				IgG concentration (BAU/ml)			MNT titer			
Disease severity	Age	Gender	n	N‐IgG	S‐IgG (RBD)	S‐IgG (SFL)	NAb wt	NAb Alpha	NAb Beta	NAb Delta
**Severe**	<60y	M+F	22	1.5 [0.88‐2.7]	3.9 [2.5‐6.1]	4.7 [3.0‐7.2]	27 [17‐41]	21 [14‐34]	8.1 [5.0‐13]	10 [7.1‐15]
		M	12	2.0 [0.99‐4.0]	4.7 [2.4‐9.0]	5.5 [2.9‐10.4]	29 [16‐55]	26 [14‐49]	9.2 [4.6‐19]	14 [8.1‐23]
		F	10	1.1 [0.45‐2.9]	3.2 [1.8‐5.7]	3.8 [2.1‐6.8]	24 [12‐47]	17 [8.5‐32]	6.8 [3.6‐13]	7.7 [4.5‐13]
	≥60y	M+F	17	1.6 [0.98‐2.5]	5.1 [3.0‐8.7]	7.6 [4.8‐12]	52 [39‐71]	30 [20‐44]	8.0 [5.1‐13]	15 [10‐22]
		M	8	0.89 [0.60‐1.3]	4.2 [2.2‐8.0]	5.8 [3.6‐9.2]	39 [27‐57]	28 [18‐42]	9.2 [4.8‐18]	13 [8.5‐21]
		F	9	2.6 [1.3‐5.0]	6.1 [2.6‐14]	9.7 [4.6‐21]	68 [45‐100]	32 [16‐61]	7.0 [3.6‐14]	16 [8.6‐31]
**Mild**	<60y	M+F	22	0.41 [0.22‐0.75]	1.6 [1.3‐2.1]	2.3 [1.9‐2.9]	15 [12‐20]	8.0 [5.4‐12]	3.6 [2.7‐4.8]	4.0 [2.8‐5.7]
		M	12	0.36 [0.14‐0.93]	1.3 [0.89‐1.8]	1.8 [1.4‐2.4]	12 [9.5‐16]	5.1 [3.1‐8.4]	2.9 [2.1‐4.1]	2.9 [2.0‐4.0]
		F	10	0.47 [0.22‐1.0]	2.2 [1.6‐3.0]	3.1 [2.3‐4.0]	20 [14‐30]	13 [8.3‐22]	4.6 [2.9‐7.3]	6.0 [3.4‐11]
	≥60y	M+F	17	0.50 [0.26‐1.1]	1.8 [1.0‐3.1]	2.1 [1.3‐3.4]	19 [11‐31]	8.5 [4.8‐15]	4.2 [2.8‐6.5]	5.6 [3.5‐8.8]
		M	8	0.94 [0.42‐2.1]	1.5 [0.72‐3.2]	1.5 [0.82‐2.8]	12 [6.1‐23]	4.6 [2.2‐9.7]	2.9 [1.8‐4.6]	4.1 [2.2‐7.6]
		F	9	0.28 [0.081‐0.98]	2.1 [0.91‐4.7]	2.9 [1.5‐5.7]	28 [14‐55]	15 [7.1‐30]	6.0 [3.2‐11]	7.4 [3.8‐14]

**Figure 4:**
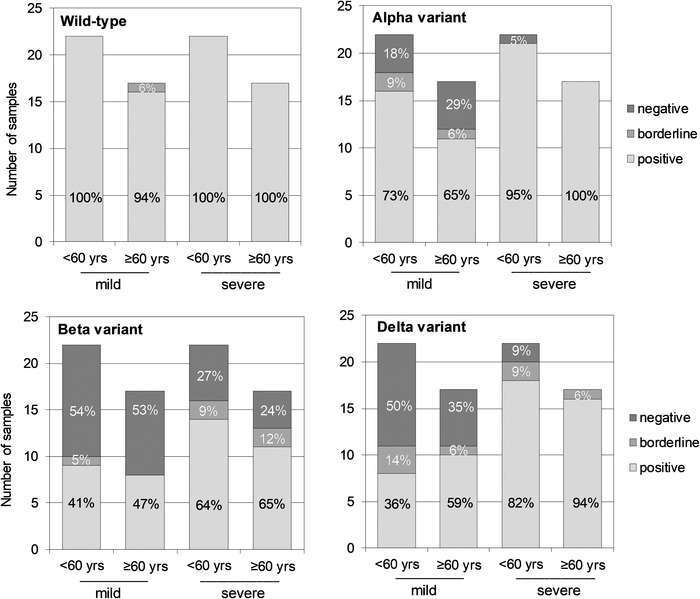
The proportion of subjects positive, low positive (borderline), and negative for neutralizing antibodies 13 months after infection against four SARS‐CoV‐2 virus strains (*n* = 78 subjects): The WT virus (B), the Alpha variant (B.1.1.7), the Beta variant (B.1.351), and the Delta variant (B.1.617.2). Each sample was tested as technical duplicates in each experiment and the experimental precision was confirmed by two control samples in each independent experiment.

For both WT virus and the Alpha variant, the proportion of seropositive individuals with severe disease remained high 13 months after infection (Fig. 4, Supporting information Table 1). Lower titers against the Alpha variant compared to the WT virus were seen in mild disease groups with an increasing proportion of low positive (borderline) or negative subjects. The greatest decrease of NAb titers was seen between the WT virus and the Beta variant with markedly lower GMTs and seropositivity with several borderline titers also in groups of severe disease. NAb titers and seropositivity for the Delta variant were also markedly lower compared to WT virus. The Delta GMT values were placed between the GMTs of the Alpha and Beta variants, yet the seropositivity of severe disease groups was relatively well preserved (≥80%) compared to that of the Beta variant (65%).

For all viruses, the subjects who recovered from the severe disease had overall 2.1 to 3.0‐fold higher NAb titers compared to those with mild disease (*p* < 0.01). The same finding was seen with all IgG concentrations. The difference in IgG concentrations between severe and mild disease was prominent in both sexes in the large study cohort (*n* = 367). However, in the small cohort (*n* = 78) only males with a mild disease had markedly lower NAb titers and S‐IgG concentrations compared to those recovered from severe disease (*p* < 0.05; Supporting information Table 2). The difference was not statistically significant for females although the trend was similar.

**Figure 5:**
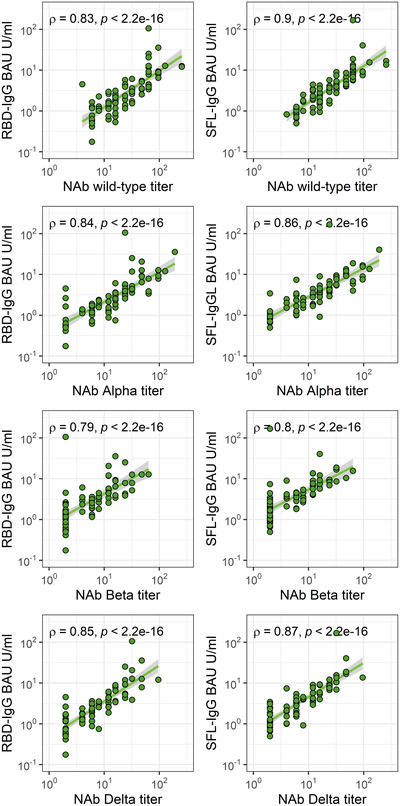
Spearman correlation (ρ) and significance (*p*) between S‐IgG antibody concentrations and neutralizing antibody (NAb) titers against the WT virus (B) and the variants of concern: Alpha (B.1.1.7), Beta (B.1.351), and Delta (B.1.617.2). One point may represent multiple samples (n = 78 subjects). Each sample was tested as technical duplicates in each experiment and the experimental precision was confirmed by two control samples in each independent experiment.

NAb titers against WT virus were higher in the elderly group (≥60 years) compared to <60 years old (*p* = 0.045) whereas NAb titers for VOCs did not differ significantly between age groups (Supporting information Table 3). We detected a strong and statistically significant correlation (*p* < 0.0001) between NAb titers and S‐IgG antibody concentrations indicating an overall parallel trend between severe and mild disease antibody levels (Fig. 5).

## Discussion

Studies of individuals who have recovered from SARS‐CoV‐2 infection are crucial in determining for how long antibodies persist after infection and whether these antibodies protect against re‐infection. We showed that S‐IgG antibodies and, most importantly, NAbs persist in most subjects for at least a year following SARS‐CoV‐2 infection. The concentration of N‐IgG, on the contrary, declined among a large proportion of subjects. In accordance with previous observations (6, 8, 31), subjects with severe infection had higher N‐IgG, S‐IgG concentrations, and NAb titers than subjects with mild infection and are expected to remain seropositive for a longer time.

Previous studies show that most patients recovering from COVID‐19 have detectable antibody responses peaking at approximately one month after infection (7, 8, 32). Antibody levels to N and S protein antigens decline during the first few months with differences in isotype and antigen specificity of the antibody (7). The decay rate has been shown to slow down thereafter (12). The relatively rapid early decline in S‐IgG antibodies followed by slower decay indicates a transition of serum antibodies from being produced by short‐lived plasmablasts to a more persistent population of long‐lived plasma cells generated later in the immune response (33). Consistently, NAbs and T cell immunity have been reported to persist at least 6 to 12 months after infection (6‐8, 11–13, 34). Our data are consistent with previous data suggesting that, even though NAb titers decline with time, NAbs persist in most subjects, at least up to 13 months.

We observed that a markedly lower proportion of subjects had N‐IgG than S‐IgG antibodies at 8 months after infection. Thereafter the concentration of N‐IgG antibodies declined to a level that was not distinguishable from unspecific, cross‐reactive antibodies among a large proportion of subjects 13 months after infection. SARS‐CoV‐2 N is produced abundantly during infection and since it is not a component in present vaccines or vaccine candidates it could potentially serve as a measure of past infection. However, our results clearly show that the sensitivity of our N‐IgG‐based antibody assay is inversely proportional to the time after infection. In agreement with our findings, the more rapid decay of N‐IgG after SARS‐CoV and SARS‐CoV‐2 infection has also been reported in other studies (32, 35, 36). The loss of sensitivity of SARS‐CoV‐2 N based antibody assays over time likely results not only from the decay of the antibodies, but from the difficulty of differentiating very low concentrations of SARS‐CoV‐2‐specific antibodies from cross‐reactive N antibodies induced by past infections with common cold human coronaviruses that share highly conserved regions (37).

Even though NAbs persist relatively long in most subjects, neutralization efficiency against the Alpha (B.1.1.7), Beta (B.1.351), and Delta (B.1.617.2) variants was decreased compared to the WT virus. This was emphasized in subjects who had recovered from mild disease representing the majority of COVID‐19 cases (1). Indeed, mild symptomatic or asymptomatic individuals may develop no or only low levels of NAbs that may wane relatively quickly after infection (38).

In line with earlier observations 9 (13) and 12 months after infection (12), we found that NAb levels against the Alpha variant were only slightly reduced, while NAb levels against the Beta variant were considerably declined compared to the WT virus. The Beta variants have been shown to evade antibody responses induced upon infection as well as vaccination (21‐23, 39, 40). Although the NAb levels were declined against the Beta variant, we observed that over 60% of hospitalized subjects were seropositive a year after infection, indicating long‐lived cross‐neutralization capacity induced by severe disease.

We detected substantially declined NAb titers against the Delta variant in subjects with mild disease, similar to what has been previously reported after vaccination or up to 12 months after SARS‐CoV‐2 infection (24, 25, 41–43). However, we observed that over 80% of the subjects who had recovered from a severe disease were seropositive against the Delta variant. This is in line with one study reporting only modestly reduced (88%) NAb levels against the Delta variant 2–4 weeks after second vaccine dose (44). Our results support the previous findings that the emerging variant Delta partially but significantly escapes NAbs (24, 25).

One previous study reported lower seropositivity rates one year after mild SARS‐CoV‐2‐infection compared to our results; 58% were positive for S1‐IgG and 85% for S‐IgG measured with enzyme immunoassay and 58% had NAb (11). Direct comparison of the IgG concentrations and NAb titers between studies may not be possible since the age groups, viruses, as well as the serological tests, differed. Neutralizing antibody tests have not been standardized and among other things, the starting dilutions of serum samples may vary between assays. The microneutralization assay used in this study utilized live virus and the starting dilution of 1:4 further enhances the sensitivity of the assay in detecting low levels of NAbs.

In our study population, we could not see a gender effect in hospitalized individuals, as previously reported (6, 31). However, hospitalized subjects ≥60 years tended to have slightly higher IgG and NAb levels compared to hospitalized subjects <60 years suggesting more severe infection in the elderly age group. Although there was no overall difference between the genders, especially males with mild disease had markedly lower NAb titers for all viruses compared to individuals who recovered from severe disease.

There is a major research effort to produce effective SARS‐CoV‐2 vaccines. The long‐term persistence of immunity after vaccination is, however, largely unknown. Evidence from convalescent sera from individuals who have recovered from infection may help determine for how long immunity persists, and whether antibodies might protect against re‐infection. Previous data shows that, when measured as IgG antibodies against S protein or RBD and NAb, immune response after two doses of SARS‐CoV‐2 vaccine is similar to that observed in convalescent sera from COVID‐19 patients (45, 46, 47, 48). Evidence of persistence of immunity after infection will help in predicting the persistence of immunity after SARS‐CoV‐2 vaccination.

We recognize certain limitations in our study. Due to high SARS‐CoV‐2 vaccine coverage in the older age groups (≥60 years of age) at the time of our study, only 11% of the participants were ≥60 years of age, the age group with the highest disease incidence and morbidity. Our results may not necessarily apply to all age groups. The number of subjects selected for the NAb titer comparison was limited but the study subjects were matched by disease severity, age, and gender, and randomly selected from the participants.

Previous studies have indicated that the presence of antibodies to SARS‐CoV‐2 was associated with a significantly reduced risk of SARS‐CoV‐2 reinfection among healthcare workers for up to 7 months after infection (27, 49). We observed that S‐IgG antibodies and NAbs persist at least a year after infection in most individuals. This strongly suggests that protection against re‐infection is long‐lived, although antibody‐mediated immunity may not persist equally well among elderly subjects. A previous study found that patients >60 years had fewer memory B cells secreting total IgG and RBD‐specific IgG than patients <60 years old 9 months after infection (9). We observed that IgG concentrations declined from 8 to 13 months more substantially in subjects ≥60 years compared to younger age groups. A similar more rapid decline in NAb concentrations was observed among the elderly compared to younger subjects who were followed up to 6 months following vaccination (50). The results of our study support previous findings indicating that protection against infection mediated by NAbs may be impaired against the VOCs, especially after a mild disease. While in the absence of NAbs reinfection is possible, cellular immunity is not similarly affected by mutations in the RBD site (22) and is likely to provide long‐term protection against severe disease.

## Materials and methods

### Study design and participants

In October 2020, 2586 subjects ≥18 years of age, native language Finnish or Swedish, living within five selected hospital districts in Finland and with a PCR‐confirmed COVID‐19 diagnosis between February 29 and April 30, 2020 were identified in the National Infectious Disease Register and invited to participate in the follow‐up study. Subjects within institutional care were excluded. Informed consent was obtained from all study subjects before sample collection. A total of 1292 (50%) subjects (median age 50.0, range 17.3‐94.3) with PCR‐confirmed COVID‐19 participated and donated a blood sample for determination of SARS‐CoV‐2 specific serum antibodies 5.9 to 9.9 months (median 7.6 months) after infection. All those previously enrolled and still living in the same hospital district (*n* = 1227) were invited to a follow‐up visit and blood sampling a year after the COVID‐19 diagnosis in March‐April 2021. By May 21, 2021, altogether 995 participants (median age 52.5, range 17.6‐95.6 years) had participated at 12.7 months (median, range 11.7 to 14.3 months) after the diagnosis of PCR‐confirmed COVID‐19. Demographics, clinical characteristics, and SARS‐CoV‐2 vaccination history of the participants were collected from the National Infectious Disease Register, the Care Register for Health Care, the Register of Primary Health Care Visits, and the National Vaccination Registry and are summarized in Table 1. The disease severity was defined as severe or mild. Severe infection was defined as an individual with laboratory‐confirmed COVID‐19 and who required hospital treatment. Mild infection was defined as an individual with laboratory‐confirmed COVID‐19 without hospital treatment. Since late December 2020 SARS‐CoV‐2 vaccinations have been offered according to the national recommendations in Finland.

### Sample processing and selection of samples

Sera were separated by centrifugation, aliquoted, and stored at ‐20°C or below. For assessment of NAbs, sera were heat‐inactivated (56°C for 30 min) and then stored at ‐20°C or below.

For assessment of persistence of serum antibodies 8 months following PCR‐confirmed COVID‐19 diagnosis, all samples taken ≤10 months after diagnosis (*n* = 1292) were selected for assessment of SARS‐CoV‐2 IgG antibody concentration and NAbs (positive/borderline/negative). For assessment of antibody persistence 13 months after infection, 400 of 995 sera were randomly selected for determination of SARS‐CoV‐2 IgG antibody concentration and NAbs. Selection criteria were: 8‐month sample available, PCR‐confirmed COVID‐19 diagnosis, no documentation of SARS‐CoV‐2 vaccination in the Register of Primary Health Care Visits by June 10^th^ 2021. Further, samples of subjects with ≥30% increase in IgG antibody concentration to both SARS‐CoV‐2 S gp antigens (full‐length spike protein (SFL) and RBD) between 8‐ and 13‐month blood sampling (n = 29) were excluded from the analysis. An additional four samples were excluded due to the late discovery of these samples not meeting selection criteria. Of the four samples, two were excluded due to vaccination and two due to samples taken >10 months after infection. Consequently, 367 sera were selected.

For comparison of NAb titers against a WT virus and VOCs (Alpha, Beta, and Delta), 80/536 13‐month sera screened to NAb (titer ≥6 against WT virus) were randomly selected as mentioned above. Later observed ≥30% increase in IgG antibody concentration between 8‐ and 13‐month samples excluded two of 80 samples, leaving total sample size to 78. SARS‐CoV‐2 IgG antibody concentration was measured from this cohort to ensure its comparability to the other 367 sera selected.

### SARS‐CoV‐2 MNT

A cytopathic effect‐based MNT was performed as previously described (51, 52). Briefly, heat‐inactivated serum samples were 2‐fold serially diluted starting from 1:4 in Eagle's MEM supplemented with penicillin, streptomycin, and 2% of heat‐inactivated fetal bovine serum. At the biosafety level 3 laboratory, pre‐titrated virus was added to obtain 100× tissue culture infectious dose 50% per well following incubation for 1 h at +37°C, 5% CO_2_. African green monkey kidney epithelial (VeroE6) cells were added and the 96‐well tissue culture plates were incubated at +37°C, 5% CO_2_ for 4 days. Wells were fixed with 30% formaldehyde and stained with crystal violet. Results were expressed as MNT titers corresponding to the reciprocal of the serum dilution that inhibited 50% of SARS‐CoV‐2 infection observed by the cytopathic effect of inoculated cells. MNT titer ≥6 was considered positive, borderline when 4, and negative when <4. Borderline values were further confirmed with biological repeats. For titer comparison, a titer of 192 was measured for the WHO International Standard (NIBSC 20/136 (53)) using the WT virus Fin1‐20.

### SARS‐CoV‐2 viruses selected for MNT

All samples were screened with WT virus Fin1‐20 (B lineage): hCoV‐19/Finland/1/2020 (GISAID accession ID EPI_ISL_407079; GenBank accession ID MZ934691) for NAb positivity. Fin1‐20 was the first SARS‐CoV‐2 strain detected in Finland in January 2020. Virus isolation and propagation were performed in Vero E6 cells (51). A smaller subset of samples was analyzed also with VOCs isolated in Finland during January 2021: Fin34‐21, Fin32‐21, and May 2021: Fin37‐21, which stand for the Alpha, Beta, and Delta variant, respectively. Alpha variant (B.1.1.7) Fin34‐21 indicates the isolate hCoV‐19/Finland/THL‐202102301/2021 (EPI_ISL_2590786; MZ944886). Spike region of the isolate hCoV‐19/Finland/THL‐202101018/2021 (Fin32‐21) showed typical Beta variant (B.1.351) amino acid changes (EPI_ISL_3471851; MZ944846). The Delta variant (B.1.617.2) Fin37‐21 indicates hCoV‐19/Finland/THL‐202117309/2021 (EPI_ISL_2557176; MZ945494). All variant viruses were isolated and propagated (passages 1–2) in VeroE6‐TMPRSS2‐H10 cells (54) and further propagated in Vero E6 cells (passage 3) for MNT.

### SARS‐CoV‐2 fluorescent multiplex immunoassay

The SARS‐CoV‐2 fluorescent multiplex immunoassay (FMIA) has been previously described in detail by Ekström et al. (52) and Solastie et al. (55). Briefly, diluted sera, reference, and controls were mixed with microspheres conjugated with SARS‐CoV‐2 N and SFL and RBD of the spike protein. IgG antibodies were detected by R‐Phycoerythrin‐conjugated secondary antibody and median fluorescence intensity was measured with MAGPIX system (Luminex) and BAU (U/ml) were interpolated from 5‐parameter logistic curves with xPONENT (v. 4.2, Luminex) created by 7‐point serial fourfold diluted reference sera calibrated against WHO International Standard (NIBSC code 20/136; (53)). When the median fluorescence intensity of a sample was below the linear range of the reference, the sample was assigned an antibody concentration half of the limit of detection (0.0094, 0.012, and 0.0057 BAU/ml for N‐, SFL‐, and RBD‐IgG). A sample was considered positive for SARS‐CoV‐2 S‐IgG when SFL and RBD specific antibody concentrations were ≥0.089 and ≥0.13 BAU/ml, respectively. A sample was considered positive for N‐IgG when N‐IgG concentration was ≥0.58 BAU/ml. The cut‐offs for seropositivity were determined during clinical validation of the FMIA and yielded both sensitivity and specificity of 100% for SFL‐ and RBD‐IgG and 98.6% and 100% for N‐IgG for samples taken 13 to 150 days post‐onset of symptoms, respectively (52, 55).

### Statistical methods

We calculated the geometric mean concentrations (GMC) and GMTs with 95% confidence intervals (CI) for IgG and NAb levels, respectively. We assessed the statistical differences in antibody levels between groups using the Kruskal‐Wallis test with Bonferroni correction. Differences in mean IgG concentrations between 8 and 13 months after infection were compared using Student's paired *t*‐test and log‐transformed data. The statistical significance level of difference was set to p<0.05. We used Spearman correlation in the correlation analyses. MNT titers <4 were assigned a titer value of 2. Samples with IgG concentrations below the limit of detection were assigned an antibody concentration equal to half of the limit of detection. Statistical analyses were performed using SPSS v27 and R (v4.0.4) with Rstudio (v1.4.1106).

## Ethics approval and patient consent statement

The study protocol was approved by the ethical committee of the Hospital District of Helsinki and Uusimaa and registered under the study protocol HUS/1137/2020. Informed consent was obtained from all study subjects before sample collection.

## Author contributions

M.M., N.E., and A.H. designed the experiments. M.M., A.A.P., and H.N. contributed to the study design. C.V. and A.S. developed and performed the FMIA tests. A.H. developed and performed the microneutralization tests. P.Ö. coordinated the virus isolations. A.H., N.E., and A.S. analyzed the data. E.I. and N.E coordinated the participant recruitment, sample collection, and sample processing. A.H., N.E., and M.M. wrote the manuscript and all co‐authors contributed to the edition of the text.

## Conflict of interest

Finnish Institute for Health and Welfare has received research funding for unrelated studies from GlaxoSmithKline Vaccines (N.E., C.V., A.A.P. and M.M. as investigators), Pfizer (A.A.P.), and Sanofi Pasteur (A.A.P.). The other authors report no potential conflicts of interest.

### Peer review

The peer review history for this article is available at https://publons.com/publon/10.1002/eji.202149535

**Abbreviations**
BAU: binding antibody unit concentration
COVID‐19: Coronavirus Disease 2019
FMIA: fluorescent multiplex immunoassay
GMC: geometric mean concentration
GMT: geometric mean titer
MNT: microneutralization test
NAb: neutralizing antibody
N: nucleoprotein
PCR: polymerase chain reaction
RBD: receptor‐binding domain
SARS‐CoV‐2: Severe acute respiratory syndrome coronavirus 2
S: spike protein
SFL: spike full length
VOC: variants of concern

## Supporting information

Supporting information

## Data Availability

The data that support the findings of this study are available from the corresponding author upon reasonable request. The complete data are not publicly available due to privacy or ethical restrictions.
