# Supplementary material for: Persistence of neutralizing antibodies a year after SARS‐CoV‐2 infection in humans
Source: Eur J Immunol. 2021 Oct 8;51(12):3202–13. doi: 10.1002/eji.202149535 (PMC8646652; doi:10.1002/eji.202149535)
Supplement: Supplementary file 1 — Supporting information [file EJI-51-3202-s001.pdf]

Supplementary Figure 1. Spearman correlation (ρ) and significance (*p*) between neutralizing antibody (NAb) titers against the wild-type virus (B) and variants of concern: Alpha (B.1.1.7), Beta (B.1.351) and Delta (B.1.617.2). One point may represent multiple samples (n=78).

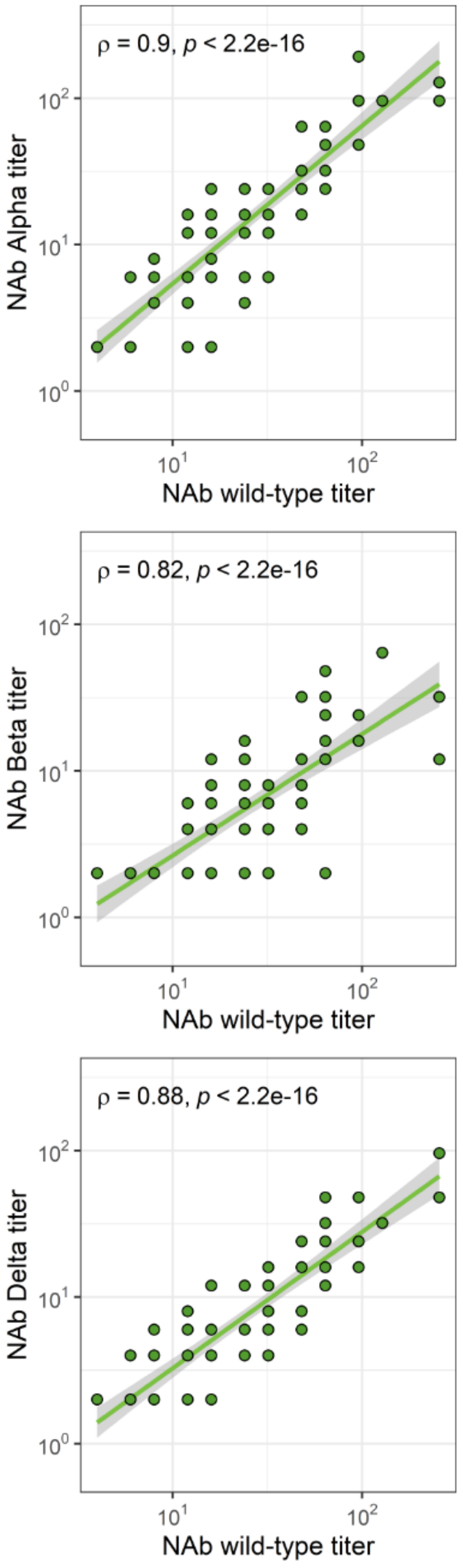

Supplementary Table 1. The proportion of subjects (M=male; F=female) positive for nucleoprotein (N) and spike proteins (SFL and RBD) IgG antibodies and the proportion of subjects positive and low positive (borderline) for neutralizing antibodies (NAb) 13 months after infection against four SARS-CoV-2 virus strains (n=78).

				**IgG positivity%**			**MNT positivity (%)**<br>***MNT borderline (%)***			
**Disease severity**	**Age**	**Gender**	**n**	**N-IgG**	**S-IgG (RBD)**	**S-IgG (SFL)**	**NAb wt**	**NAb Alpha**	**NAb Beta**	**NAb Delta**
**Severe**	<60y	M+F	22	18/22 (82)	22/22 (100)	22/22 (100)	22/22 (100)	21/22 (95)	14/22 (64)<br>*2/22 (9)*	18/22 (82)<br>*2/22 (9)*
		M	12	10/12 (83)	12/12 (100)	12/12 (100)	12/12 (100)	12/12 (100)	8/12 (67)	10/12 (83)<br>*2/12 (17)*
		F	10	8/10 (80)	10/10 (100)	10/10 (100)	10/10 (100)	9/10 (90)	6/10 (60)<br>*2/10 (20)*	8/10 (80)
	≥60v	M+F	17	14/17 (82)	17/17 (100)	17/17 (100)	17/17 (100)	17/17 (100)	11/17 (65)<br>*2/17 (12)*	16/17 (94)<br>*1/17 (6)*
		M	8	6/8 (75)	8/8 (100)	8/8 (100)	8/8 (100)	8/8 (100)	5/8 (63)<br>*2/8 (25)*	8/8 (100)
		F	9	8/9 (89)	9/9 (100)	9/9 (100)	9/9 (100)	9/9 (100)	6/9 (67)	8/9 (89)<br>*1/9 (11)*
**Mild**	<60v	M+F	22	11/22 (50)	22/22 (100)	22/22 (100)	22/22 (100)	16/22 (73)<br>*2/22 (9)*	9/22 (41)<br>*1/22 (5)*	8/22 (37)<br>*3/22 (14)*
		M	12	6/12 (50)	12/12 (100)	12/12 (100)	12/12 (100)	6/12 (50)<br>*2/12 (17)*	3/12 (25)<br>*1/12 (8)*	2/12 (17)<br>*2/12 (17)*
		F	10	5/10 (50)	10/10 (100)	10/10 (100)	10/10 (100)	10/10 (100)	6/10 (60)	6/10 (60)<br>*1/10 (10)*
	≥60v	M+F	17	9/17 (53)	17/17 (100)	17/17 (100)	16/17 (94)<br>*1/17 (6)*	11/17 (65)<br>*1/17 (6)*	8/17 (47)	10/17 (59)<br>*1/17 (6)*
		M	8	6/8 (75)	8/8 (100)	8/8 (100)	7/8 (88)<br>*1/8 (13)*	3/8 (38)<br>*1/8 (13)*	2/8 (25)	3/8 (38)<br>*1/8 (13)*
		F	9	3/9 (33)	9/9 (100)	9/9 (100)	9/9 (100)	8/9 (89)	6/9 (67)	7/9 (78)

Supplementary Table 2. Influence of gender and disease severity 13 months after infection on spike protein IgG concentrations (SFL, RBD) and neutralizing antibody titers against wild-type (B) virus and variant viruses Alpha (B.1.1.7), Beta (B.1.351) and Delta (B.1.617.2) (n=78). Kruskal-Wallis with Bonferroni correction, adjusted p-values. P-values <0.05 are marked in bold.

Compared groups		Wild-type virus (B)	Alpha variant (B.1.1.7)	Beta variant (B.1.351)	Delta variant (B.1.617.2)	IgG-SFL	IgG-RBD
Male mild	Male severe	**0.0029**	**0.000033**	**0.0013**	**0.000045**	**0.00022**	**0.0024**
Female mild	Female severe	0.51	1.00	1.00	0.82	0.29	0.41
Female mild	Male mild	0.19	**0.047**	0.34	0.15	0.28	0.55
Female severe	Male severe	1.00	1.00	1.00	1.00	1.00	1.00
Female severe	Male mild	**0.0006**	**0.00041**	**0.033**	**0.0011**	**0.00041**	**0.0024**
Female mild	Male severe	1.00	0.40	0.48	0.18	0.22	0.42

Supplementary Table 3. Influence of gender, age and disease severity 13 months after infection on spike protein IgG concentrations (SFL, RBD) and neutralizing antibody titers against wild type (B) virus and variant viruses Alpha (B.1.1.7), Beta (B.1.351) and Delta (B.1.617.2) (n=78). Kruskal-Wallis with Bonferroni correction, adjusted p-values. P-values <0.05 are marked in bold.

	Wild-type virus (B)	Alpha variant (B.1.1.7)	Beta variant (B.1.351)	Delta variant (B.1.617.2)	IgG-SFL	IgG-RBD
**Gender**	0.066	0.13	0.47	0.27	0.18	0.22
**Age (<60 vs ≥60 yrs)**	**0.045**	0.33	0.61	0.14	0.33	0.39
**Disease severity**	**0.00022**	**0.000030**	**0.0011**	**0.000022**	**0.000015**	**0.00014**
